# Supplementary material for: Systematic review of oral health in slums and non-slum urban settings of Low and Middle-Income Countries (LMICs): Disease prevalence, determinants, perception, and practices
Source: PLoS One. 2024 Nov 8;19(11):e0309319. doi: 10.1371/journal.pone.0309319 (PMC11548750; doi:10.1371/journal.pone.0309319)
Supplement: S1 Appendix — (DOCX) [file pone.0309319.s001.docx]

S1 Appendix: Details of web address and link for each database search strategy details and results for all electronic databases and journals and grey literature

| **Data**  **base** | **Link** | **Search** | **Date** | **Hits** |
| --- | --- | --- | --- | --- |
| **PUBMED** | <https://pubmed.ncbi.nlm.nih.gov/advanced/>  <https://pubmed.ncbi.nlm.nih.gov/?term=longquerya8ad9b1abbe2d97a69c9&size=200> | Search: (((((((((dental caries) OR (periodontal disease)) OR (oral cancer)) OR (dental fluorosis)) OR (dental trauma)) OR (dental erosion)) AND ((((((((dental health survey or oral health survey) OR (prevalence or incidence)) OR (risk factors or associated factors)) OR (oral health or hygiene practices)) OR (oral health or hygiene practices)) OR (interviews or qualitative research or focus group discussions)) OR (oral health beliefs or myths or attitudes or knowledge)) OR (oral health service utilization or access to oral health services))) AND (((poverty area) OR (slum or suburb or sub-urban or favela or baladi or bandas de mieri or barraco or ghetto or downtown or informal settlement or barrio marginal or barrio or bidonville or barek or inner city or busbee or challis or shack or cherek bete or d'agata or outskirts or ester or galdos or gecekondu or ghettos or krishi or shah or kalyan or katsas or lobban or loteamento or medina chouaib or morro or mudun safi or shantytown or musique or shanty town or solares or tanaka or saudis or tugurio or usuku or modolo or watts or zopadpattis)) OR (non-slum or urban non slum or metropolis or township or municipal or municipality or civic or city or city centre or city center))) AND (((((developing country) OR (Third world country)) OR (LMIC)) OR (Low and middle income country)) OR (Africa or Asia or Caribbean or West Indies or South America or Latin America or Central America))) AND ((English[Language]) AND (("2000/01/10"[Date - Publication] : "2023/06/11"[Date - Publication]))) | 11/ 06 /2023 | 2,071 |
| **Embase** | OVID  https://ovidsp.dc1. ovid.com/sp-4.06  .0a/ ovidweb.cgi | **Database: Embase Classic+Embase <1947 to 2023 June 23> Search Strategy:**  --------------------------------------------------------------------------------  1     exp dental caries/ or dental caries*.mp. (69262)  2     exp periodontal disease/ or exp periodontitis/ or exp gingivitis/ or periodontal disease*.mp. (131975)  3     exp mouth cancer/ or oral cancer*.mp. or exp mouth tumor/ (155397)  4     exp dental fluorosis/ or fluorosis/ or dental fluorosis*.mp. (5420)  5     exp tooth injury/ or exp tooth fracture/ or dental trauma*.mp. (14289)  6     dental erosion*.mp. (1545)  7     health survey/ or oral health survey*.mp. (233192)  8     oral disease epidemiology*.mp. or exp mouth disease/ or exp epidemiology/ (5218407)  9     prevalence*.mp. or exp prevalence/ (1412047)  10     incidence*.mp. or exp incidence/ (1627511)  11     exp risk factor/ or risk factors*.mp. (1638640)  12     exp dental care/ or attitude to health/ or oral health practice*.mp. or exp mouth hygiene/ (368428)  13     dental prevention/ or prevention/ or prevention*.mp. or exp tertiary prevention/ or exp secondary prevention/ or exp caries prevention/ or exp primary prevention/ (2250243)  14     exp semi structured interview/ or exp interview/ or interview*.mp. or exp structured interview/ or exp unstructured interview/ (669063)  15     exp qualitative research/ or qualitative research*.mp. (132028)  16     exp health care utilization/ or exp "facilities and services utilization"/ or utilization*.mp. (495239)  17     exp health care access/ or health service access*.mp. (103095)  18     exp developing country/ or developing country*.mp. (108529)  19     (Afghanistan or Albania or Algeria or Angola or Antigua or Barbuda or Argentina or Armenia or Armenian or Aruba or Azerbaijan or Bahrain or Bangladesh or Barbados or Benin or Byelarus or Byelorussian or Belarus or Belorussian or Belorussia or Belize or Bhutan or Bolivia or Bosnia or Herzegovina or Hercegovina or Botswana or Brasil or Brazil or Bulgaria or Burkina Faso or Burkina Fasso or Upper Volta or Burundi or Urundi or Cambodia or Khmer Republic or Kampuchea or Cameroon or Cameroons or Cameron or Camerons or Cape Verde or Central African Republic or Chad or Chile or China or Colombia or Comoros or Comoro Islands or Comores or Mayotte or Congo or Zaire or Costa Rica or Cote d'Ivoire or Ivory Coast or Croatia or Cuba or Cyprus or Czechoslovakia or Czech Republic or Slovakia or Slovak Republic or Djibouti or French Somaliland or Dominica or Dominican Republic or East Timor or East Timur or Timor Leste or Ecuador or Egypt or United Arab Republic or El Salvador or Eritrea or Estonia or Ethiopia or Fiji or Gabon or Gabonese Republic or Gambia or Gaza or Georgia Republic or Georgian Republic or Ghana or Gold Coast or Greece or Grenada or Guatemala or Guinea or Guam or Guiana or Guyana or Haiti or Honduras or Hungary or India or Maldives or Indonesia or Iran or Iraq or Isle of Man or Jamaica or Jordan or Kazakhstan or Kazakh or Kenya or Kiribati or Korea or Kosovo or Kyrgyzstan or Kirghizia or Kyrgyz Republic or Kirghiz or Kirgizstan or Lao PDR or Laos or Latvia or Lebanon or Lesotho or Basutoland or Liberia or Libya or Lithuania or Macedonia or Madagascar or Malagasy Republic or Malaysia or Malaya or Malay or Sabah or Sarawak or Malawi or Nyasaland or Mali or Malta or Marshall Islands or Mauritania or Mauritius or Agalega Islands or Mexico or Micronesia or Middle East or Moldova or Moldovia or Moldovian or Mongolia or Montenegro or Morocco or Ifni or Mozambique or Myanmar or Myanma or Burma or Namibia or Nepal or Netherlands Antilles or New Caledonia or Nicaragua or Niger or Nigeria or Northern Mariana Islands or Oman or Muscat or Pakistan or Palau or Palestine or Panama or Paraguay or Peru or Philippines or Philipines or Phillipines or Phillippines or Poland or Portugal or Puerto Rico or Romania or Rumania or Roumania or Russia or Russian or Rwanda or Ruanda or Saint Kitts or St Kitts or Nevis or Saint Lucia or St Lucia or Saint Vincent or St Vincent or Grenadines or Samoa or Samoan Islands or Navigator Island or Navigator Islands or Sao Tome or Saudi Arabia or Senegal or Serbia or Montenegro or Seychelles or Sierra Leone or Slovenia or Sri Lanka or Ceylon or Solomon Islands or Somalia or South Africa or Sudan or Suriname or Surinam or Swaziland or Syria or Tajikistan or Tadzhikistan or Tadjikistan or Tadzhik or Tanzania or Thailand or Togo or Togolese Republic or Tonga or Trinidad or Tobago or Tunisia or Turkey or Turkmenistan or Turkmen or Uganda or Ukraine or Uruguay or USSR or Soviet Union or Union of Soviet Socialist Republics or Uzbekistan or Uzbek or Vanuatu or New Hebrides or Venezuela or Vietnam or Viet Nam or West Bank or Yemen or Yugoslavia or Zambia or Zimbabwe or Rhodesia).mp. (3044628)  20     (Africa or Asia or Caribbean or West Indies or South America or Latin America or Central America).mp. (451837)  21     third world country*.mp. (224)  22     exp lowest income group/ or exp low income country/ or income*.mp. or exp middle income country/ or exp middle income group/ (275773)  23     LMIC*.mp. (13363)  24     exp poverty/ or slum*.mp. (61845)  25     (suburb or sub-urban or favela or baladi or bandas de mieri or barraco or ghetto or downtown or informal settlement or barrio marginal or barrio or bidonville or barek or inner city or busbee or challis or shack or cherek bete or d'agata or outskirts or ester or galdos or gecekondu or ghettos or krishi or shah or kalyan or katsas or lobban or loteamento or medina chouaib or morro or mudun safi or shantytown or musique or shanty town or solares or tanaka or saudis or tugurio or usuku or modolo or watts or zopadpattis).mp. (389940)  26     (non-slum or urban non slum or metropolis or township or municipal or municipality or civic or city or city centre or city center).mp. (298965)  27     1 or 2 or 3 or 4 or 5 or 6 (359193)  28     7 or 8 or 9 or 10 (6171573)  29     11 or 12 or 13 or 14 or 15 or 16 or 17 (4974424)  30     28 or 29 (9453904)  31     18 or 19 or 20 or 21 or 22 or 23 (3465083)  32     24 or 25 or 26 (730900)  33     27 and 30 and 31 and 32 (2321)  34     limit 33 to (english language and yr="2020 -Current") (1803) | 24/ 06/ 2023 | 1,803 |
| **SCOPUS** | Accessed through the University of Warwick Library Service  <https://warwick.ac.uk/services/library/> | TITLE-ABS-KEY(dental or tooth or teeth or caries or oral or mouth or periodontal or periodontitis or gingivitis) AND (prevalence or incidence or survey or epidemiology or "risk factor" or interview or questionnaire or "focus group" or qualitative or perception or belief or attitude or knowledge or myth or access or utilization or practice or facility or prevention or hygiene) AND (Afghanistan or Albania or Algeria or Angola or Antigua or Barbuda or Argentina or Armenia or Armenian or Aruba or Azerbaijan or Bahrain or Bangladesh or Barbados or Benin or Belarus or Byelorussian or Belarus or Belorussian or Belorussia or Belize or Bhutan or Bolivia or Bosnia or Herzegovina or Hercegovina or Botswana or Brasil or Brazil or Bulgaria or "Burkina Faso" or "Burkina Fasso" or "Upper Volta" or Burundi or Urundi or Cambodia or "Khmer Republic" or Kampuchea or Cameroon or Cameroons or Cameron or Camerons or "Cape Verde" or "Central African Republic" or Chad or Chile or China or Colombia or Comoros or "Comoro Islands" or Comores or Mayotte or Congo or Zaire or "Costa Rica" or "Cote d'Ivoire" or "Ivory Coast" or Croatia or Cuba or Cyprus or Czechoslovakia or "Czech Republic" or Slovakia or "Slovak Republic" or Djibouti or "French Somaliland" or Dominica or "Dominican Republic" or "East Timor" or "East Timur" or "Timor Leste" or Ecuador or Egypt or "United Arab Republic" or "El Salvador" or Eritrea or Estonia or Ethiopia or Fiji or Gabon or "Gabonese Republic" or Gambia or Gaza or "Georgia Republic" or "Georgian Republic" or Ghana or "Gold Coast" or Greece or Grenada or Guatemala or Guinea or Guam or Guiana or Guyana or Haiti or Honduras or Hungary or India or Maldives or Indonesia or Iran or Iraq or "Isle of Man" or Jamaica or Jordan or Kazakhstan or Kazakh or Kenya or Kiribati or Korea or Kosovo or Kyrgyzstan or Kirghizia or "Kyrgyz Republic" or Kirghiz or Kirgizstan or "Lao PDR" or Laos or Latvia or Lebanon or Lesotho or Basutoland or Liberia or Libya or Lithuania or Macedonia or Madagascar or "Malagasy Republic" or Malaysia or Malaya or Malay or Sabah or Sarawak or Malawi or Nyasaland or Mali or Malta or "Marshall Islands" or Mauritania or Mauritius or "Agalega Islands" or Mexico or Micronesia or "Middle East" or Moldova or Moldovia or Moldovian or Mongolia or Montenegro or Morocco or Ifni or Mozambique or Myanmar or Myanma or Burma or Namibia or Nepal or "Netherlands Antilles" or "New Caledonia" or Nicaragua or Niger or Nigeria or "Northern Mariana Islands" or Oman or Muscat or Pakistan or Palau or Palestine or Panama or Paraguay or Peru or Philippines or Philipines or Phillipines or Phillippines or Poland or Portugal or "Puerto Rico" or Romania or Rumania or Roumania or Russia or Russian or Rwanda or Ruanda or "Saint Kitts" or "St Kitts" or Nevis or "Saint Lucia" or "St Lucia" or "Saint Vincent" or "St Vincent" or Grenadines or Samoa or "Samoan Islands" or "Navigator Island" or "Navigator Islands" or "Sao Tome" or "Saudi Arabia" or Senegal or Serbia or Montenegro or Seychelles or "Sierra Leone" or Slovenia or "Sri Lanka" or Ceylon or "Solomon Islands" or Somalia or "South Africa" or Sudan or Suriname or Surinam or Swaziland or Syria or Tajikistan or Tadzhikistan or Tadjikistan or Tadzhik or Tanzania or Thailand or Togo or "Togolese Republic" or Tonga or Trinidad or Tobago or Tunisia or Turkey or Turkmenistan or Turkmen or Uganda or Ukraine or Uruguay or USSR or "Soviet Union" or "Union of Soviet Socialist Republics" or Uzbekistan or Uzbek or Vanuatu or "New Hebrides" or Venezuela or Vietnam or "Viet Nam" or "West Bank" or Yemen or Yugoslavia or Zambia or Zimbabwe or Rhodesia or Africa or Asia or Caribbean or "West Indies" or "South America" or "Latin America" or "Central America" or "developing country" or "third world country" or "low and middle income country" or "low income country" or "middle income country" or LMIC or LMICs) AND (slum or favela or baladi or "bandas de mieri" or barraco or ghetto or "informal settlement" or "barrio marginal" or barrio or bidonville or barek or busbee or challis or shack or "cherek bete" or d'agata or outskirts or ester or galdos or gecekondu or ghettos or krishi or shah or kalyan or katsas or lobban or loteamento or "medina chouaib" or morro or "mudun safi" or shantytown or musique or "shanty town" or solares or tanaka or saudis or tugurio or usuku or modolo or watts or zopadpattis or metropolis or township or municipal or municipality or urban or city) AND ( LIMIT-TO ( SUBJAREA,"DENT" ) )  Search retrieved 6855 records without limiting to subject area of dentistry – included mostly irrelevant records  Search retrieved 605 records after applying the limit to subject area of dentistry | 10/ 03/ 2024 | 605 |
| **Web of Science** | Accessed through the University of Warwick Library Service  <https://warwick.ac.uk/services/library/> | # Web of Science Search Strategy (v0.1)  # Database: Web of Science Core Collection  # Entitlements:  - WOS.SCI: 1970 to 2024  - WOS.AHCI: 1975 to 2024  - WOS.ESCI: 2015 to 2024  - WOS.ISTP: 1990 to 2024  - WOS.SSCI: 1900 to 2024  - WOS.ISSHP: 1990 to 2024  # Searches:  1: TI=(dental or tooth or teeth or caries or oral or mouth or periodontal or periodontitis or gingivitis) Results: 490935  2: AB=(dental or tooth or teeth or caries or oral or mouth or periodontal or periodontitis or gingivitis) Results: 900781  3: TI=(prevalence or incidence or survey or epidemiology or risk factor or interview or questionnaire or focus group or qualitative or perception or belief or attitude or knowledge or myth or access or utilization or practice or facility or prevention or hygiene)  Results: 2901004  4: AB=(prevalence or incidence or survey or epidemiology or risk factor or interview or questionnaire or focus group or qualitative or perception or belief or attitude or knowledge or myth or access or utilization or practice or facility or prevention or hygiene) Results: 10083311  5: TI=(Afghanistan or Albania or Algeria or Angola or Antigua or Barbuda or Argentina or Armenia or Armenian or Aruba or Azerbaijan or Bahrain or Bangladesh or Barbados or Benin or Belarus or Byelorussian or Belarus or Belorussian or Belorussia or Belize or Bhutan or Bolivia or Bosnia or Herzegovina or Hercegovina or Botswana or Brasil or Brazil or Bulgaria or Burkina Faso or Burkina Fasso or Upper Volta or Burundi or Urundi or Cambodia or Khmer Republic or Kampuchea or Cameroon or Cameroons or Cameron or Camerons or Cape Verde or Central African Republic or Chad or Chile or China or Colombia or Comoros or Comoro Islands or Comores or Mayotte or Congo or Zaire or Costa Rica or Cote d'Ivoire or Ivory Coast or Croatia or Cuba or Cyprus or Czechoslovakia or Czech Republic or Slovakia or Slovak Republic or Djibouti or French Somaliland or Dominica or Dominican Republic or East Timor or East Timur or Timor Leste or Ecuador or Egypt or United Arab Republic or El Salvador or Eritrea or Estonia or Ethiopia or Fiji or Gabon or Gabonese Republic or Gambia or Gaza or Georgia Republic or Georgian Republic or Ghana or Gold Coast or Greece or Grenada or Guatemala or Guinea or Guam or Guiana or Guyana or Haiti or Honduras or Hungary or India or Maldives or Indonesia or Iran or Iraq or Isle of Man or Jamaica or Jordan or Kazakhstan or Kazakh or Kenya or Kiribati or Korea or Kosovo or Kyrgyzstan or Kirghizia or Kyrgyz Republic or Kirghiz or Kirgizstan or Lao PDR or Laos or Latvia or Lebanon or Lesotho or Basutoland or Liberia or Libya or Lithuania or Macedonia or Madagascar or Malagasy Republic or Malaysia or Malaya or Malay or Sabah or Sarawak or Malawi or Nyasaland or Mali or Malta or Marshall Islands or Mauritania or Mauritius or Agalega Islands or Mexico or Micronesia or Middle East or Moldova or Moldovia or Moldovian or Mongolia or Montenegro or Morocco or Ifni or Mozambique or Myanmar or Myanma or Burma or Namibia or Nepal or Netherlands Antilles or New Caledonia or Nicaragua or Niger or Nigeria or Northern Mariana Islands or Oman or Muscat or Pakistan or Palau or Palestine or Panama or Paraguay or Peru or Philippines or Philipines or Phillipines or Phillippines or Poland or Portugal or Puerto Rico or Romania or Rumania or Roumania or Russia or Russian or Rwanda or Ruanda or Saint Kitts or St Kitts or Nevis or Saint Lucia or St Lucia or Saint Vincent or St Vincent or Grenadines or Samoa or Samoan Islands or Navigator Island or Navigator Islands or Sao Tome or Saudi Arabia or Senegal or Serbia or Montenegro or Seychelles or Sierra Leone or Slovenia or Sri Lanka or Ceylon or Solomon Islands or Somalia or South Africa or Sudan or Suriname or Surinam or Swaziland or Syria or Tajikistan or Tadzhikistan or Tadjikistan or Tadzhik or Tanzania or Thailand or Togo or Togolese Republic or Tonga or Trinidad or Tobago or Tunisia or Turkey or Turkmenistan or Turkmen or Uganda or Ukraine or Uruguay or USSR or Soviet Union or Union of Soviet Socialist Republics or Uzbekistan or Uzbek or Vanuatu or New Hebrides or Venezuela or Vietnam or Viet Nam or West Bank or Yemen or Yugoslavia or Zambia or Zimbabwe or Rhodesia or Africa or Asia or Caribbean or West Indies or South America or Latin America or Central America or developing country or third world country or low and middle income country or low income country or middle income country or LMIC or LMICs) Results: 3016575  6: AB=(Afghanistan or Albania or Algeria or Angola or Antigua or Barbuda or Argentina or Armenia or Armenian or Aruba or Azerbaijan or Bahrain or Bangladesh or Barbados or Benin or Belarus or Byelorussian or Belarus or Belorussian or Belorussia or Belize or Bhutan or Bolivia or Bosnia or Herzegovina or Hercegovina or Botswana or Brasil or Brazil or Bulgaria or Burkina Faso or Burkina Fasso or Upper Volta or Burundi or Urundi or Cambodia or Khmer Republic or Kampuchea or Cameroon or Cameroons or Cameron or Camerons or Cape Verde or Central African Republic or Chad or Chile or China or Colombia or Comoros or Comoro Islands or Comores or Mayotte or Congo or Zaire or Costa Rica or Cote d'Ivoire or Ivory Coast or Croatia or Cuba or Cyprus or Czechoslovakia or Czech Republic or Slovakia or Slovak Republic or Djibouti or French Somaliland or Dominica or Dominican Republic or East Timor or East Timur or Timor Leste or Ecuador or Egypt or United Arab Republic or El Salvador or Eritrea or Estonia or Ethiopia or Fiji or Gabon or Gabonese Republic or Gambia or Gaza or Georgia Republic or Georgian Republic or Ghana or Gold Coast or Greece or Grenada or Guatemala or Guinea or Guam or Guiana or Guyana or Haiti or Honduras or Hungary or India or Maldives or Indonesia or Iran or Iraq or Isle of Man or Jamaica or Jordan or Kazakhstan or Kazakh or Kenya or Kiribati or Korea or Kosovo or Kyrgyzstan or Kirghizia or Kyrgyz Republic or Kirghiz or Kirgizstan or Lao PDR or Laos or Latvia or Lebanon or Lesotho or Basutoland or Liberia or Libya or Lithuania or Macedonia or Madagascar or Malagasy Republic or Malaysia or Malaya or Malay or Sabah or Sarawak or Malawi or Nyasaland or Mali or Malta or Marshall Islands or Mauritania or Mauritius or Agalega Islands or Mexico or Micronesia or Middle East or Moldova or Moldovia or Moldovian or Mongolia or Montenegro or Morocco or Ifni or Mozambique or Myanmar or Myanma or Burma or Namibia or Nepal or Netherlands Antilles or New Caledonia or Nicaragua or Niger or Nigeria or Northern Mariana Islands or Oman or Muscat or Pakistan or Palau or Palestine or Panama or Paraguay or Peru or Philippines or Philipines or Phillipines or Phillippines or Poland or Portugal or Puerto Rico or Romania or Rumania or Roumania or Russia or Russian or Rwanda or Ruanda or Saint Kitts or St Kitts or Nevis or Saint Lucia or St Lucia or Saint Vincent or St Vincent or Grenadines or Samoa or Samoan Islands or Navigator Island or Navigator Islands or Sao Tome or Saudi Arabia or Senegal or Serbia or Montenegro or Seychelles or Sierra Leone or Slovenia or Sri Lanka or Ceylon or Solomon Islands or Somalia or South Africa or Sudan or Suriname or Surinam or Swaziland or Syria or Tajikistan or Tadzhikistan or Tadjikistan or Tadzhik or Tanzania or Thailand or Togo or Togolese Republic or Tonga or Trinidad or Tobago or Tunisia or Turkey or Turkmenistan or Turkmen or Uganda or Ukraine or Uruguay or USSR or Soviet Union or Union of Soviet Socialist Republics or Uzbekistan or Uzbek or Vanuatu or New Hebrides or Venezuela or Vietnam or Viet Nam or West Bank or Yemen or Yugoslavia or Zambia or Zimbabwe or Rhodesia or Africa or Asia or Caribbean or West Indies or South America or Latin America or Central America or developing country or third world country or low and middle income country or low income country or middle income country or LMIC or LMICs) Results: 3786041  7: TI=(slum or favela or baladi or bandas de mieri or barraco or ghetto or informal settlement or barrio marginal or barrio or bidonville or barek or busbee or challis or shack or cherek bete or d'agata or outskirts or ester or galdos or gecekondu or ghettos or krishi or shah or kalyan or katsas or lobban or loteamento or medina chouaib or morro or mudun safi or shantytown or musique or shanty town or solares or tanaka or saudis or tugurio or usuku or modolo or watts or zopadpattis or metropolis or township or municipal or municipality or urban or city) Results: 654491  8: AB=(slum or favela or baladi or bandas de mieri or barraco or ghetto or informal settlement or barrio marginal or barrio or bidonville or barek or busbee or challis or shack or cherek bete or d'agata or outskirts or ester or galdos or gecekondu or ghettos or krishi or shah or kalyan or katsas or lobban or loteamento or medina chouaib or morro or mudun safi or shantytown or musique or shanty town or solares or tanaka or saudis or tugurio or usuku or modolo or watts or zopadpattis or metropolis or township or municipal or municipality or urban or city) Results: 1305420  9: #1 or #2 Results: 1118729  10: #3 or #4 Results: 11415313  11: #5 or #6 Results: 5195646  12: #7 or #8 Results: 1591649  13: #9 and #10 and #11 and #12 Results: 6895  14: #9 and #10 and #11 and #12 Timespan: 2000-01-01 to 2024-03-10 Results: 6609 | 10/ 03/ 2024 | 6,609 |
| **HINARI** | <https://login.research4life.org/tacsgr0extranet_who_int/hinari/en/journals.php> | ((LMIC) OR (developing countries)) AND ((slum or poverty areas) OR (suburb or sub-urban or favela or baladi or bandas de mieri or barraco or ghetto or downtown or informal settlement or barrio marginal or barrio or bidonville or barek or inner city or busbee or challis or shack or cherek bete or d'agata or outskirts or ester or galdos or gecekondu or ghettos or krishi or shah or kalyan or katsas or lobban or loteamento or medina chouaib or morro or mudun safi or shantytown or musique or shanty town or solares or tanaka or saudis or tugurio or usuku or modolo or watts or zopadpattis) OR (non-slum or urban non slum or metropolis or township or municipal or municipality or civic or city or city centre or city center)) AND ((oral health survey) OR (oral disease epidemiology) OR (prevalence or incidence) OR (risk factors) OR (oral health practice) OR (oral health service utilization) OR (oral health behaviour) OR (qualitative research) OR (qualitative interview)) | 11/ 06/ 2023 | 635 |
| **Medline** | <https://ovidsp.dc1.ovid.com/sp-4.07.0b/ovidweb.cgi> | **Database: Ovid MEDLINE(R) ALL <1946 to June 22, 2023>** Search Strategy:  --------------------------------------------------------------------------------  1     exp DMF Index/ or exp Dental Caries/ or dental caries*.mp. (59197)  2     exp Alveolar Bone Loss/ or exp Periodontal Diseases/ or periodontal disease*.mp. or exp Gingivitis/ or exp Periodontitis/ (103964)  3     exp Precancerous Conditions/ or exp Mouth Neoplasms/ or oral cancer*.mp. (138820)  4     exp Fluorosis, Dental/ or dental fluorosis*.mp. (3183)  5     exp Tooth Fractures/ or exp Tooth Injuries/ or exp Tooth Avulsion/ or dental trauma*.mp. (11980)  6     exp Tooth Erosion/ or dental erosion*.mp. (3490)  7     exp Dental Health Surveys/ or dental health survey*.mp. or exp Oral Health/ (40800)  8     exp Prevalence/ or prevalence*.mp. (882995)  9     exp Incidence/ or incidence*.mp. (1042125)  10     exp Risk Factors/ or risk factors*.mp. (1247903)  11     exp Health Knowledge, Attitudes, Practice/ or exp Oral Hygiene/ or exp Dental Care/ or oral health practice*.mp. (178212)  12     exp Tertiary Prevention/ or exp Secondary Prevention/ or prevention*.mp. or exp Primary Prevention/ (2000041)  13     exp Qualitative Research/ or interviews*.mp. (303173)  14     exp Focus Groups/ or focus group*.mp. (69440)  15     exp Culture/ or beliefs*.mp. (241210)  16     myths*.mp. (6980)  17     exp "Patient Acceptance of Health Care"/ or health care utilization*.mp. (179534)  18     exp Health Services Accessibility/ or health service access*.mp. (134762)  19     developing country*.mp. or exp Developing Countries/ (86400)  20     (Afghanistan or Albania or Algeria or Angola or Antigua or Barbuda or Argentina or Armenia or Armenian or Aruba or Azerbaijan or Bahrain or Bangladesh or Barbados or Benin or Byelarus or Byelorussian or Belarus or Belorussian or Belorussia or Belize or Bhutan or Bolivia or Bosnia or Herzegovina or Hercegovina or Botswana or Brasil or Brazil or Bulgaria or Burkina Faso or Burkina Fasso or Upper Volta or Burundi or Urundi or Cambodia or Khmer Republic or Kampuchea or Cameroon or Cameroons or Cameron or Camerons or Cape Verde or Central African Republic or Chad or Chile or China or Colombia or Comoros or Comoro Islands or Comores or Mayotte or Congo or Zaire or Costa Rica or Cote d'Ivoire or Ivory Coast or Croatia or Cuba or Cyprus or Czechoslovakia or Czech Republic or Slovakia or Slovak Republic or Djibouti or French Somaliland or Dominica or Dominican Republic or East Timor or East Timur or Timor Leste or Ecuador or Egypt or United Arab Republic or El Salvador or Eritrea or Estonia or Ethiopia or Fiji or Gabon or Gabonese Republic or Gambia or Gaza or Georgia Republic or Georgian Republic or Ghana or Gold Coast or Greece or Grenada or Guatemala or Guinea or Guam or Guiana or Guyana or Haiti or Honduras or Hungary or India or Maldives or Indonesia or Iran or Iraq or Isle of Man or Jamaica or Jordan or Kazakhstan or Kazakh or Kenya or Kiribati or Korea or Kosovo or Kyrgyzstan or Kirghizia or Kyrgyz Republic or Kirghiz or Kirgizstan or Lao PDR or Laos or Latvia or Lebanon or Lesotho or Basutoland or Liberia or Libya  or Lithuania or Macedonia or Madagascar or Malagasy Republic or Malaysia or Malaya or Malay or Sabah or Sarawak or Malawi or Nyasaland or Mali or Malta or Marshall Islands or Mauritania or Mauritius or Agalega Islands or Mexico or Micronesia or Middle East or Moldova or Moldovia or Moldovian or Mongolia or Montenegro or Morocco or Ifni or Mozambique or Myanmar or Myanma or Burma or Namibia or Nepal or Netherlands Antilles or New Caledonia or Nicaragua or Niger or  Nigeria or Northern Mariana Islands or Oman or Muscat or Pakistan or Palau or Palestine or Panama or Paraguay or Peru or Philippines or Philipines or Phillipines or Phillippines or Poland or Portugal or Puerto Rico or Romania or Rumania or Roumania or Russia or Russian or Rwanda or Ruanda or Saint Kitts or St Kitts or Nevis or Saint Lucia or St Lucia or Saint Vincent or St Vincent or Grenadines or Samoa or Samoan Islands or Navigator Island or Navigator Islands or Sao Tome or Saudi Arabia or Senegal or Serbia or Montenegro or Seychelles or Sierra Leone or Slovenia or Sri Lanka or Ceylon  or Solomon Islands or Somalia or South Africa or Sudan or Suriname or Surinam or Swaziland or Syria or Tajikistan or Tadzhikistan or Tadjikistan or Tadzhik or Tanzania or Thailand or Togo or Togolese Republic or Tonga or Trinidad or Tobago or Tunisia or Turkey or Turkmenistan or Turkmen or Uganda or Ukraine or Uruguay or USSR or Soviet Union or Union of Soviet Socialist Republics or Uzbekistan or Uzbek or Vanuatu or New Hebrides or Venezuela or Vietnam or Viet Nam or West Bank or Yemen or Yugoslavia or Zambia or Zimbabwe or Rhodesia).mp. (2298554)  21     (Africa or Asia or Caribbean or West Indies or South America or Latin America or Central America).mp. (344488)  22     third world country*.mp. (134)  23     LMIC*.mp. (10498)  24     (low and middle income countries*).mp. (30182)  25     exp Poverty Areas/ or slum*.mp. (9880)  26     (suburb or sub-urban or favela or baladi or bandas de mieri or barraco or ghetto or downtown or informal settlement or barrio marginal or barrio or bidonville or barek or inner city or busbee or challis or shack or cherek bete or d'agata or outskirts or ester or galdos or gecekondu or ghettos or krishi or shah or kalyan or katsas or lobban or loteamento or medina chouaib or morro or mudun safi or shantytown or musique or shanty town or solares or tanaka or saudis or tugurio or usuku or modolo or watts or zopadpattis).mp. (155346)  27     (non-slum or urban non slum or metropolis or township or municipal or municipality or civic or city or city centre or city center).mp. (211089)  28     1 or 2 or 3 or 4 or 5 or 6 (305244)  29     7 or 8 or 9 or 10 (2721898)  30     11 or 12 or 13 or 14 or 15 or 16 or 17 or 18 (2803944)  31     29 or 30 (4981092)  32     19 or 20 or 21 or 22 or 23 or 24 (2474915)  33     25 or 26 or 27 (364163)  34     28 and 31 and 32 and 33 (1278)  35     limit 34 to (english language and yr="2020 -Current") (855)*********************** | 24/ 06/ 2023 | 855 |
| **CRD DARE Database** | <https://www.crd.york.ac.uk/CRDWeb/HistoryPage.asp> | 1 (developing country) IN DARE FROM 2000 TO 2023 15  2 (third world country) IN DARE FROM 2000 TO 2023 0  3 (low and middle income country) IN DARE FROM 2000 TO 2023 9  4 (slum) IN DARE FROM 2000 TO 2023 3  5 (suburb or sub-urban or favela or baladi or bandas de mieri or barraco or ghetto or downtown or informal settlement or barrio marginal or barrio or bidonville or barek or inner city or busbee or challis or shack or cherek bete or d'agata or outskirts or ester or galdos or gecekondu or ghettos or krishi or shah or kalyan or katsas or lobban or loteamento or medina chouaib or morro or mudun safi or shantytown or musique or shanty town or solares or tanaka or saudis or tugurio or usuku or modolo or watts or zopadpattis) IN DARE FROM 2000 TO 2023 324  6 (Africa or Asia or Caribbean or West Indies or South America or Latin America or Central America) IN DARE FROM 2000 TO 2023 668  7 (non-slum or urban non slum or metropolis or township or municipal or municipality or civic or city or city centre or city center) IN DARE FROM 2000 TO 2023 72  8 (dental caries) IN DARE FROM 2000 TO 2023 125  9 (periodontal disease) IN DARE FROM 2000 TO 2023 44  10 (oral cancer) IN DARE FROM 2000 TO 2023 26  11 (dental fluorosis) IN DARE FROM 2000 TO 2023 4  12 (dental trauma) IN DARE FROM 2000 TO 2023 4  13 (dental erosion) IN DARE FROM 2000 TO 2023 2  14 (oral health survey) IN DARE FROM 2000 TO 2023 0  15 (health survey) IN DARE FROM 2000 TO 2023 49  16 (oral disease epidemiology) IN DARE FROM 2000 TO 2023 0  17 (disease epidemiology) IN DARE FROM 2000 TO 2023 63  18 (prevalence) IN DARE FROM 2000 TO 2023 1296  19 (incidence) IN DARE FROM 2000 TO 2023 3546  20 (risk factors) IN DARE FROM 2000 TO 2023 3295  21 (health care practice) IN DARE FROM 2000 TO 2023 16  22 (health care beliefs) IN DARE FROM 2000 TO 2023 0  23 (beliefs) IN DARE FROM 2000 TO 2023 89  24 (myths) IN DARE FROM 2000 TO 2023 3  25 (practices) IN DARE FROM 2000 TO 2023 326  26 (interview) IN DARE FROM 2000 TO 2023 206  27 (qualitative research) IN DARE FROM 2000 TO 2023 160  28 (prevention) IN DARE FROM 2000 TO 2023 7948  29 (utilization) IN DARE FROM 2000 TO 2023 527  30 (access) IN DARE FROM 2000 TO 2023 580  31 #1 OR #2 OR #3 OR #6 685  32 #4 OR #5 OR #7 390  33 #8 OR #9 OR #10 OR #11 OR #12 OR #13 193  34 #14 OR #15 OR #16 OR #17 OR #18 OR #19 OR #20 OR #21 OR #22 OR #23 OR #24 OR #26 OR #27 OR #28 OR #29 OR #30 13777  35 #31 OR #32 1067  36 #33 AND #34 AND #35 8 | 11/ 06/ 2023 | 8 |
| **Essential Health Links** | https://www.hivsharespace.net/resource-library | Oral health survey <https://www.hivsharespace.net/search?search_api_fulltext=oral+health+survey&type=All&sort_by=created&sort_order=DESC&x=0&y=0>  Oral OR dental health survey <https://www.hivsharespace.net/search?search_api_fulltext=oral+health+survey&type=All&sort_by=created&sort_order=DESC&x=0&y=0>  No dental health related topics on the page | 11/ 06/ 2023 | 29  No result found |
| **African Index Medicus (AIM)** | <https://indexmedicus.afro.who.int/aim/opac_css/index.php?lvl=more_results&autolevel1=1> | 532 electronic documents or title(s) found 'oral or dental health survey OR beliefs OR myths OR practices OR utilization of dental services | 09/ 06/ 2023 | 532 |
| **BabelMeSH** | <https://babelmesh.nlm.nih.gov/search/search.php?from=eng&com=> | **oral** OR **dental** **health** **survey** AND **slums** | 09/ 06/ 2023 | 556 |
| **ELDIS** | <https://www.eldis.org/search?query=oral+OR+dental+health+survey+OR+practices+OR+beliefs+OR+myths+OR+utilization+of+dental+facility&theme=C563&country=A1159&object_type%5BDocument%5D=Document&region=&publication_year_from=2000&publication_year_to=2023&publisher=&perpage=100&sort=publication_date_desc&op=Go&form_build_id=form-YbbTEpT62zcwnbdP3ALEflu6s3PfeU_GHQAXHQjnrkY&form_id=idscontent_form> | No dental health related fields in the Focus Topics  Outputs are country specific  Searching documents for '**oral OR dental health survey OR practices OR beliefs OR myths OR utilization of dental facility'** with a thematic focus on **Health** in Nigeria | 09/ 06/ 2023 | 0 |
| **Bioline International** | <http://www.bioline.org.br/search?search_target=all_pub&search_string=oral+or+dental+health+survey> | oral or dental health survey OR beliefs OR myths OR practices OR utilization of dental services  oral, dental, health, survey, slum  oral or health survey  slum | 09/ 06/ 2023 | 0  0  17  71 |
| **Reference checking** |  |  |  | 2 |
| **contacting experts in the field** |  |  |  | 2 |
| **studies from conference abstracts** |  |  |  | 1 |
|  |  |  | **Total** | 13,796 |
|  |  |  | **After de- duplication** | 7,908 |
