# Supplementary material for: Systematic review of oral health in slums and non-slum urban settings of Low and Middle-Income Countries (LMICs): Disease prevalence, determinants, perception, and practices
Source: PLoS One. 2024 Nov 8;19(11):e0309319. doi: 10.1371/journal.pone.0309319 (PMC11548750; doi:10.1371/journal.pone.0309319)
Supplement: S2 Appendix — (DOCX) [file pone.0309319.s002.docx]

**S3 Appendix:** **Deviation from the originally registered protocol**

| **Section** | **Previous statement in the original protocol** | **Change introduced** | **New statement** | **Justification** |
| --- | --- | --- | --- | --- |
| Participant/ Population  (Exclusion criterion) | Mentally challenged, disabled, and institutionalized adult population groups as well as children population | Adjusted | Mentally challenged, disabled, and institutionalized adult population groups as well as children-only population | Many study populations cut across children and adult age groups, which still provide relevant information |
|  | Children population less than 18 years | Adjusted | Children-only population less than 18 years |  |
| Type of study (exclusion criteria) | Study publications in which data for the urban population are compared with the rural population as opposed to the slum population | Deleted | N/A | Searches yielded very few hits of studies focusing exclusively on slum populations |
|  | Study publications from general urban settings in LMICs without specific reference to data from slum and non-slum population | Deleted | N/A |  |
| Type of study (inclusion criteria) | N/A | New statement added | Quantitative studies carried out in a representative urban population or national surveys covering such (slum) populations. | Searches yielded few slum-specific studies and therefore it was considered useful to expand the coverage to quantitative studies that were likely to have included some slum population |
|  | N/A | New statement added | Qualitative studies carried out in slums or other similar urban settings (e.g. poverty areas within cities) | Searches yield few slum-specific studies and therefore it was considered useful to expand the coverage to qualitative studies that were carried out in other urban settings similar to slums |
|  | | | | |
