# Supplementary material for: Systematic review of oral health in slums and non-slum urban settings of Low and Middle-Income Countries (LMICs): Disease prevalence, determinants, perception, and practices
Source: PLoS One. 2024 Nov 8;19(11):e0309319. doi: 10.1371/journal.pone.0309319 (PMC11548750; doi:10.1371/journal.pone.0309319)
Supplement: S3 Appendix — (DOCX) [file pone.0309319.s003.docx]

**S4 Appendix: Distribution of included studies according to continents, countries and settings**

| **S/N** | **Continent** | **Country** | **Setting(s) included / compared** | **Author** | **Year** | **Participants** |
| --- | --- | --- | --- | --- | --- | --- |
|  | Asia | India | Urban / Urban slum | Patel et al | 2017 | People >10 years of age. (N = 300) |
|  | Asia | Bangladesh | Urban slum | Hannan et al | 2014 | Participants of all age groups and sex (N = 3,904) |
|  | Asia | India | Urban slum | Airen et al. | 2014 | Age range 5 – 64.  (N = 143) |
|  | Asia | Pakistan | Urban slum | Habib et al. | 2022 | Age range 20 – 50 years  (N = 385) |
|  | Asia | India | Urban slum clusters | Chakraborti et al | 2023 | Adults ages 18 years and above  (N= 210) |
|  | Asia | India | Urban / Rural | Handa et al. | 2016 | WHO's index ages and age groups of 5, 12, 15, 35-44, and 65-74 years (N = 810) |
|  | Asia | Nepal | Disadvantaged population | Singh et al. | 2020 | Population of Foklyan area, Dharan, Nepal: 5 years 12 years 15 years 35–44 years 65–74 years (N = 310) |
|  | Asia | China | Urban / Rural | Wang  et al | 2002 | Whole population sample (N = 140,712) |
|  | Asia | China | Urban / Rural | Sun et al | 2018 | Adult population 34 – 44 years of age (N = 4,410) |
|  | Asia | Malaysia | Urban disadvantaged / National population | Jaafar et al. | 2014 | Adults 19 years & > household residents (N = 586) |
|  | Asia | Iran | Urban / Rural | Hessari et al | 2006 | All 35- to 44-year-old Iranians living in Iran. (N = 8,301) |
|  | Asia | Iran | Urban | Rezaei et al | 2017 | Household head or 18 years and above (N = 894) |
|  | Asia | Iran | High and low socioeconomic regions | Gholami et al | 2012 | Adult residents 18 years and >.  FGDs conducted with a total of 46 participants |
|  | Africa | South Africa | a nationally representative population | Olutola and Ayo-Yusuf | 2012 | Adults (≥16 years) (N = 2,907) |
|  | Africa | Malawi | Whole country | Msyamboza et al. | 2016 | Ages 12, 15, 35–44 and 65–74-year-olds. (N = 5,400) |
|  | Africa | Nigeria | Whole country | Olusile et al | 2014 | Ages 18 to 81 (N = 7,630) |
|  | Africa | Nigeria | Urban / Rural | Tobin and Ajayi | 2017 | WHO index ages 5–6, 12 and 35–44 years’ age groups  (N = 150) |
|  | Africa | Nigeria | Slum versus Non slum | Osuh et al | 2022 | Adults 18 years and above  (N = 1,357) |
|  | Africa | Ghana | Metropolis/ Municipal/ district/ Urban/ Rural | Hewlett et al. | 2022 | Population in GAR comprising adults aged 25 years and above  (N= 729) |
|  | Africa | Rwanda | Urban / Rural | Morgan et al. | 2018 | Whole country 2–5, 6–11, 12–19, 20–39, and 40 and above years (N = 2,097) |
|  | Africa | Tanzania | Rural / Urban | Masalu et al | 2009 | Adult respondents from the six geographic zones of the mainland (N = 1,759) |
|  | Africa | Burtkina Faso | Urban and rural | Varenne et al.  2004 | 2004 | 6 years (n = 424), 12 years (n = 505), 18 years (n = 492) and 35–44 years (n = 493)  (N = 1,914) |
|  | South America | Brazil | Urban | Costa et al. | 2012 | Adults 35 to 44 years of age  (N = 1,150) |
