# Supplementary material for: Systematic review of oral health in slums and non-slum urban settings of Low and Middle-Income Countries (LMICs): Disease prevalence, determinants, perception, and practices
Source: PLoS One. 2024 Nov 8;19(11):e0309319. doi: 10.1371/journal.pone.0309319 (PMC11548750; doi:10.1371/journal.pone.0309319)
Supplement: S1 Table — (DOCX) [file pone.0309319.s004.docx]

**S2 Tables. Supporting tables including list of studies evaluated at full-text screening stage, an example of original data extraction form and completed risk of bias assessments for studies providing evidence for individual review sub-questions**

**S2 Table 1. The full list of included studies and studies excluded at full-text screening stage and reason(s) for exclusion**

| **S/N** | **Study** | **Full citation** | **Inclusion/ exclusion** | **Reason(s) for exclusion** |
| --- | --- | --- | --- | --- |
|  | Patel et al. 2017 | Comparative study of oral hygienic practices and oral health status among people residing in urban and urban slum of Ahmedabad municipal corporation*.* Int J. Community Med. Public Health. 2017;4(6):2181-2185. | Included | NA |
|  | Hannan et al. 2014 | Prevalence of Gingivitis, Plaque accumulation and Decayed, Missing and Filled Teeth among slum population in Bangladesh*.* Bangladesh Medical Research Council Bulletin. 2014;40(2):47-51. | Included | N/A |
|  | Osuh et al. 2022 | Prevalence and determinants of oral health conditions and treatment needs among slum and non-slum urban residents: Evidence from Nigeria*.* PLOS Global Public Health. 2022;2(4):e0000297. | Included | NA |
|  | Airen et al. 2014 | Dentition status and treatment need in urban slum dwellers in Indore city, Central India*.* Journal of Indian Association of Public Health Dentistry. 2014;12(3):163-166. | Included | N/A |
|  | Habib et al. 2022 | Oral Health Status and Oral Hygiene Practices among Urban Slum Dwellers in Rawalpindi, Islamabad, Pakistan: Oral Health and Hygiene Practices Among Urban Slum Dwellers*.* Pakistan Journal of Health Sciences. 2022:114-118. | Included | N/A |
|  | Chakraborty et al. 2023 | Oral Morbidity Pattern and its Behavioural Determinants among Adults of Urban Slums of Siliguri, India*.* JOURNAL OF CLINICAL AND DIAGNOSTIC RESEARCH. 2023;17(1):LC01-LC05. | Included | N/A |
|  | Rezaei et al. 2018 | Dental health-care service utilisation and its determinants in West Iran: a cross-sectional study*.* Int Dent J. 2018;68(3):176-182. | Included | N/A |
|  | Costa et al. 2012 | The severity of dental caries in adults aged 35 to 44 years residing in the metropolitan area of a large city in Brazil: a cross-sectional study*.* BMC Oral Health. 2012;12(1):1-11. | Included | N/A |
|  | Hewlett et al. 2022 | Assessment of oral health status in a Ghanaian population: rationale, methods, and population characteristics*.* BMC Oral Health. 2022;22(1):67. | Included | N/A |
|  | Wang et al. 2002 | The second national survey of oral health status of children and adults in China*.* International Dental Journal. 2002;52(4):283-290. | Included | N/A |
|  | Tobin and Ajayi, 2017 | Common oral conditions and correlates: an oral health survey in Kwara State Nigeria. BMC research notes. 2017;10:568. | Included | N/A |
|  | Handa et al. 2016 | Oral Health Status of Rural and Urban Population of Gurgaon Block, Gurgaon District Using WHO Assessment Form through Multistage Sampling Technique*.* Journal of clinical and diagnostic research: JCDR. 2016; Journal of Clinical and Diagnostic Research(5):ZC43-ZC51. | Included | N/A |
|  | Morgan et al. 2018 | Building oral health research infrastructure: the first national oral health survey of Rwanda*.* Global Health Action. 2018;11(1):1477249. | Included | N/A |
|  | Sun et al. 2018 | The prevalence and associated factors of periodontal disease among 35 to 44-year-old Chinese adults in the 4th National Oral Health Survey*.* Chin J Dent Res. 2018;21(4):241-247. | Included | N/A |
|  | Masalu et al. 2009 | Oral health related behaviors among adult Tanzanians: a national pathfinder survey*.* BMC Oral Health. 2009;9(1):1-9. | Included | N/A |
|  | Hessari et al. 2007 | Oral health among 35-to 44-year-old Iranians*.* Medical Principles and Practice. 2007;16(4):280-285. | Included | N/A |
|  | Varenne et al. 2004 | Oral health status of children and adults in urban and rural areas of Burkina Faso, Africa*.* International dental journal. 2004;54(2):83-89. | Included | N/A |
|  | Olutola and Ayo-Yusuf. 2012 | Socio-environmental factors associated with self-rated oral health in South Africa: a multilevel effects model*.* International journal of environmental research and public health. 2012;9(10):3465-3483. | Included | N/A |
|  | Msyamboza et al. 2016 | Magnitude of dental caries, missing and filled teeth in Malawi: National Oral Health Survey*.* BMC Oral Health. 2016;16(1):1-6. | Included | N/A |
|  | Olusile et al. 2014 | Self-rated oral health status, oral health service utilization, and oral hygiene practices among adult Nigerians. BMC Oral Health. 2014;14:140. | Included | N/A |
|  | Jaafar et al. 2014 | Is the burden of oral diseases higher in urban disadvantaged community compared to the national prevalence? BMC Public Health. 2014;14(3):S2. | Included | N/A |
|  | Singh et al. 2020 | Assessment of oral health status and treatment needs among people of Foklyan area, Dharan, Nepal*.* BMC Oral Health. 2020;20(1):320. | Included | N/A |
|  | Gholami et al. 2012 | Common Perceptions of Periodontal Health and Illness among Adults: A Qualitative Study*.* ISRN Dentistry. 2012;2012:671879. | Included | N/A |
|  | Snyder et al. 2017 | Differences in the prevalence of non-communicable disease between slum dwellers and the general population in a large urban area in Brazil. Tropical medicine and infectious disease. 2017 Sep 16;2(3):47. | Excluded | Did not cover dental health |
|  | Kyobutungi et al, 2008 | The burden of disease profile of residents of Nairobi's slums: Results from a Demographic Surveillance System. Population health metrics. 2008 Dec;6:1-8.: Results from a Demographic Surveillance System | Excluded | Did not cover dental health/ oral diseases |
|  | Asiki et al, 2021 | Trends and risk factors for non-communicable diseases mortality in Nairobi slums (2008–2017). Global Epidemiology. 2021 Nov 1;3:100049. | Excluded | Did not cover dental health |
|  | Abedassar et al 2022. | Oral health status and oral hygiene behaviour of orphan children: a survey in support centers in Kerman City, Iran, in 2019. *Journal of Oral Health and Oral Epidemiology*, *11*(1), 32-39. | Excluded | Children population |
|  | Lo et al, 2000 | Oral health status and treatment need of 11-13-year-old urban children in Tibet, China. *Community Dental Health*, *17*(3), 161-164. | Excluded | Children population |
|  | Aboutalebi, et al. 2022 | Assessment of decayed, missing, and filled teeth and the influencing factors in 7-10-year-old students in Quchan, Iran, 2020-2021. *Journal of Oral Health and Oral Epidemiology*, *11*(2), 81-87. | Excluded | Children only |
|  | Petersen et al, 2001 | Oral health status and oral health behaviour of urban and rural schoolchildren in Southern Thailand. *International dental journal*, *51*(2), 95-102. | Excluded | Children only population |
|  | Yotat et al. 2015 | Oral health status of the elderly at Tonga, West Region, Cameroon. *International journal of dentistry*, *2015*(1), 820416. | Excluded | Only elderly population |
|  | Abuhaloob and Petersen, 2021 | Oral health status and oral health behaviour among 5-to 6-year-old Palestinian schoolchildren–towards engagement of parents and schoolteachers for oral health through schools. *Oral Health and Preventive Dentistry*, *19*(1), 673-682. | Excluded | Children only |
|  | Achmad et al, 2020 | Analysis of Dental Caries & Gingivitis with the Occurrence of Stunting in Children in Makassar City (Tamalanrea Subdistrict). *Systematic Reviews in Pharmacy*, *11*(4). | Excluded | Children only |
|  | Al-Haddad et al, 2010 | Oral health status and treatment needs among school children in Sana’a City, Yemen. *International journal of dental hygiene*, *8*(2), 80-85. | Excluded | Children only |
|  | Bruce et al, 2002 | Oral health status of peri-urban schoolchildren in Accra, Ghana. *International Dental Journal*, *52*(4), 278-282. | Excluded | Children |
|  | Nnawuihe et al, 2021 | Dental caries experience and oral health behaviour; A survey of a Nigerian rural adult population. *African Journal of Oral Health*, *11*(1), 22-29. | Excluded | Strictly rural communities |
|  | Bogale et al, 2021 | Dental caries experience and associated factors in adults: a cross-sectional community survey within Ethiopia. *BMC public health*, *21*, 1-12. | Excluded | Majorly rural |
|  | Lawal et al, 2013 | Oral health practices of adult inhabitants of a traditional community in Ibadan, Nigeria. *Nigerian Journal of Medicine*, *22*(3), 212-217. | Excluded | Rural |
|  | Rwenyonyi et al, 2010 | Assessment of factors associated with dental caries in rural communities in Rakai District, Uganda. *Clinical oral investigations*, *15*, 75-80. | Excluded | Rural |
|  | Kumar et al. 2016 | Assessment of oral health status and treatment needs of Santhal tribes of Dhanbad District, Jharkhand. *Journal of International Society of Preventive and Community Dentistry*, *6*(4), 338-343. | Excluded | Villages |
|  | Kassim et al, 2006 | Oral health status among Kenyans in a rural arid setting: dental caries experience and knowledge on its causes. *East African medical journal*, *83*(2), 100-105. | Excluded | Rural |
|  | Gudsoorkar, 2022 | *Exploration of oral hygiene practices, oral health status, and related quality of life of individuals residing in the Burere, Nyambogo and Roche villages of the Rorya district of Tanzania, East Africa: A mixed-methods study* (Master's thesis, University of Cincinnati). | Excluded | Villages |
|  | Adeniyi et al, 2012 | An appraisal of the oral health care system in Nigeria. *International Dental Journal*, *62*(6), 292-300. | Excluded | Commentary |
|  | Agrawal et al, 2011 | Oral hygiene and periodontal status among detainees in a juvenile detention center, India. *Oral health & preventive dentistry*, *9*(3). | Excluded | Institutionalised -participant population |
|  | Károlyházy et al. 2003. | Dental status and oral health of patients with epilepsy: an epidemiologic study. *Epilepsia*, *44*(8), 1103-1108. | Excluded | Institutionalised / patient population |
|  | Chaudhary et al. 2019. | Dental health status and oral health behaviours of patients with facial burn in Pakistan. *BMC Oral Health*, *19*, 1-10. | Excluded | Institutionalised / Patient population |
|  | Al-Mobeeriek, 2012 | Oral health status among psychiatric patients in Riyadh, Saudi Arabia. *West indian medical journal*, *61*(5). | Excluded | Institutionalised / patient population |
|  | Shekarchizadeh et al. 2019 | Oral health status and its determinants among opiate dependents: a cross-sectional study. *BMC Oral Health*, *19*,1-7. | Excluded | Patient population |
|  | Al-Badr et al, 2021 | Dental caries prevalence among Type 1 diabetes mellitus (T1DM) 6-to 12-year-old children in Riyadh, Kingdom of Saudi Arabia compared to non-diabetic children. *The Saudi Dental Journal*, *33*(5), 276-282. | Excluded | Institutionalised / patient population |
|  | Ahmed et al, 2023 | Assessment of oral health knowledge, attitude, practice and dmft scores among patients at King Faisal University, Al-Ahsa. *Medicina*, *59*(4), 688. | Excluded | Institutionalised / patient population |
|  | Rao et al, 2017 | Oral health status and treatment needs of Gunj marketing yard laborers of Raichur City, Karnataka. *Journal of Pharmacy and Bioallied Sciences*, *9*(3), 195-200. | Excluded | Institutionalised- participant population |
|  | Varenne et al, 2006 | Illness-related behaviour and utilization of oral health services among adult city-dwellers in Burkina Faso: evidence from a household survey. *BMC health services research*, *6*, 1-11. | Excluded | Companion paper |
|  | Mosha et al, 2005 | Oral health status and treatment needs among Tanzanians of different age groups. Tanzania Dental Journal. 2005;12(1):18-27. | Excluded | Companion paper |
|  | Sun et al, 2020 | Prevalence and associated factors of periodontal conditions among 55-to 74-year-old adults in China: results from the 4th National Oral Health Survey. Clinical Oral Investigations. 2020 Dec;24:4403-12. | Excluded | Companion paper |
|  | Hau et al, 2017 | Oral health status and possible explanatory factors of an inner-city low-income community. *Journal of Dental Sciences*, *12*(1), 49-55. | Excluded | High income country, Canada |
|  | Akbar et al, 2019 | Relationship between health service access to dental conditions in urban and rural areas in Indonesia. *Pesquisa Brasileira em Odontopediatria e Clínica Integrada*, *19*, e4652. | Excluded | Pilot survey |

**S2 Table 2. An example of initial data extraction form to determine which review sub-questions a study was eligible for**

| **S/N** | **Study’s full citation** | **Setting(s) included/ compared** | **Methods** | **Outcome**  **Measures** | **Applicable sub-review questions** | **Data Extractor** | **Date of data extraction** |
| --- | --- | --- | --- | --- | --- | --- | --- |
| 1 | Patel, A. B., Shah, R. R., & Ramanuj, V. B. (2017). Comparative study of oral hygienic practices and oral health status among people residing in urban and urban slum of Ahmedabad municipal corporation. Int J Community Med Public Health, 4(6), 2181-5. | Urban / Urban slum | Purposely selected residents of Municipal Corporation (AMC) area of Vejalpur ward, Ahmedabad.  1. Questionnaire  2. WHO oral health assessment (1997) | Dental caries, gum disease- bleeding gums, dental stains, dental abscess, mouth ulcers, halitosis, tooth sensitivity, tonsilitis malocclusion,  dental hygiene practices | **a b, d** | **MO** | **June – September 2020** |
|  | Patel, A. B., Shah, R. R., & Ramanuj, V. B. (2017). Comparative study of oral hygienic practices and oral health status among people residing in urban and urban slum of Ahmedabad municipal corporation. Int J Community Med Public Health, 4(6), 2181-5. | Urban / Urban slum | Non-random selection of residents of participants  Questionnaire and World Health Organization (WHO) oral health surveys and assessment methods  (1997) | Dental caries, staining, abscess formation, mouth ulcers, bad breath, gingivitis, tooth sensitivity, malocclusion, tonsillitis, bleeding gums  dental hygiene practices | **a b, d** | **TST** |  |
| 2 | Osuh, M. E., Oke, G. A., Lilford, R. J., Owoaje, E., Harris, B., Taiwo, O. J., ... & Chen, Y. F. (2022). Prevalence and determinants of oral health conditions and treatment needs among slum and non-slum urban residents: Evidence from Nigeria. PLOS Global Public Health, 2(4), e0000297. | Urban / Urban slum | Multistage stratified random sampling of adult residents of slum and non-slum  Questionnaire and  WHO oral health assessment (2013) | Dental caries,  Periodontal disease  Dental erosion  Dental trauma  Oral mucosal lesions  Enamel fluorosis  Denture use  Treatment needed  Slum vs non-slum sites  Disease determinants  Perception, Dental health practices, Utilisation of dental services | **a, b, c, d, e** | **MO** | **July- August 2023** |
|  | Osuh, M. E., Oke, G. A., Lilford, R. J., Owoaje, E., Harris, B., Taiwo, O. J., ... & Chen, Y. F. (2022). Prevalence and determinants of oral health conditions and treatment needs among slum and non-slum urban residents: Evidence from Nigeria. PLOS Global Public Health, 2(4), e0000297. | Urban / Urban slum | Multistage stratified random sampling of adult residents of slum and non-slum  Questionnaire  WHO oral health assessment methods (2013) | Caries, Perio. dx,  Erosion, trauma  Oral mucosal lesions, fluorosis, dental treatment reqd. oral dx determinants, perception and practices about oral health and dental service utilisation  Slum vs non-slum sites | **a, b, c, d, e** | **TST** |  |

**S2 Table 3. MMAT scores for studies included in sub-review question 1 - Prevalence of oral diseases among adult residents of slum and urban settings of LMICs**

|  | **Methodological quality** | Patel et al 2017 [20] | Osuh et al. 2022. [4] | Hannan et al. 2014  [21] | Airen et al. 2014 [22] | Habib et al. 2022 [23] | Chakraborti et al 2023[24] | Singh  et al. 2020 [40] | Costa et al. 2012  [26] | Hewlett et al. 2022  [27] | Jaafar et al. 2014  [39] | Msyamboza  et al. 2016 [37] | Morgan et al. 2018  [31] | Wang et al. 2002 [28] | Tobin and Ajayi 2017 [29] | Handa et al. 2016 [30] | Sun et al.  2018 [32] | Hess ari et al. 2007 [34] | Varenne et al. 2004 [35] |
| --- | --- | --- | --- | --- | --- | --- | --- | --- | --- | --- | --- | --- | --- | --- | --- | --- | --- | --- | --- |
| **SCREENING QUESTIONS FOR ALL TYPES OF STUDY DESIGNS** | S1. Are there clear research questions? | Y | Y | Y | Y | Y | Y | Y | Y | Y | Y | Y | Y | Y | Y | Y | Y | Y | Y |
|  | S2. Do the collected data allow to address the research questions? | Y | Y | Y | Y | Y | Y | Y | Y | Y | Y | Y | Y | Y | Y | Y | Y | Y | Y |
| **QUANTITATIVE DESCRIPTIVE STUDIES** | 4.1. Is the sampling strategy relevant to address the research question? | Y | Y | Y | Y | Can’t tell | Y | Y | Y | Y | Y | Y | Y | Y | Y | Y | Y | Y | Y |
|  | 4.2. Is the sample representative of the target population? | N | Y | Y | Can’t tell | Can’t tell | Y | Y | Y | Y | Y | Y | Y | Y | N | Y | Y | Y | Y |
|  | 4.3. Are the measurements appropriate? | Y | Y | Y | Y | Y | Y | Y | Y | Y | Y | Y | Y | Y | Y | Y | Y | Y | Y |
|  | 4.4. Is the risk of nonresponse bias low? | Can’t tell | Y | Can’t tell | Y | Can't tell | Y | Y | Y | Y | Y | Y | Y | Y | Can’t tell | Y | Y | Y | Can’t tell |
|  | 4.5. Is the statistical analysis appropriate to answer the research question? | Y | Y | Y | Y | Y | Y | Y | Y | Y | Y | Y | Y | Y | Y | Y | Y | Y | Y |

**S2 Table 4. MMAT scores for studies included in sub-review question 2 - Associated factors/ risk factors of oral diseases in adults residing in slum and urban settings of LMICs**

|  | **Methodological quality** | Patel et al 2017 [20] | Osuh et al. 2022. [4] | Hannan et al. 2014  [21] | Airen et al. 2014 [22] | Chakraborti et al 2023[24] | Costa et al. 2012  [26] | Hewlett et al. 2022  [27] | Jaafar et al. 2014  [39] | Msyamboza  et al. 2016 [37] | Morgan et al. 2018  [31] | Wang et al. 2002 [28] | Tobin and Ajayi 2017 [29] | Sun et al.  2018 [32] | Hess ari et al. 2007 [34] |
| --- | --- | --- | --- | --- | --- | --- | --- | --- | --- | --- | --- | --- | --- | --- | --- |
| **Screening questions for all types of study designs** | S1. Are there clear research questions? | Y | Y | Y | Y | Y | Y | Y | Y | Y | Y | Y | Y | Y | Y |
|  | S2. Do the collected data allow to address the research questions? | Y | Y | Y | Y | Y | Y | Y | Y | Y | Y | Y | Y | Y | Y |
| **QUANTITATIVE DESCRIPTIVE STUDIES** | 4.1. Is the sampling strategy relevant to address the research question? | Y | Y | Y | Y | Y | Y | Y | Y | Y | Y | Y | Y | Y | Y |
|  | 4.2. Is the sample representative of the target population? | N | Y | Y | Y | Y | Y | Y | Y | Y | Y | Y | N | Y | Y |
|  | 4.3. Are the measurements appropriate?s | Y | Y | Y | Y | Y | Y | Y | Y | Y | Y | Y | Y | Y | Y |
|  | 4.4. Is the risk of nonresponse bias low? | Can’t tell | Y | Can’t tell | Can’t tell | Y | Y | Y | Y | Y | Y | Y | Can’t tell | Y | Y |
|  | 4.5. Is the statistical analysis appropriate to answer the research question? | Y | Y | Y | Y | Y | Y | Y | Y | Y | Y | Y | Y | Y | Y |

**S2 Table 5. MMAT scores for studies included in sub-review question 3 - Perceptions of adult residents of slums and other non-slum settings of LMICs about their oral health state**

|  | **Methodological quality** | Osuh et al. 2022. [4] | Singh  et al. 2020 [40] | Gholami et al. 2012[41] | Olusile et al. 2014  [38] | Olutola & Ayo-Yusuf. 2012 [36] | Rezaei et al. 2018 [25] |
| --- | --- | --- | --- | --- | --- | --- | --- |
| **SCREENING QUESTIONS FOR ALL TYPES OF STUDY DESIGNS** | S1. Are there clear research questions? | Y | Y | Y | Y | Y | Y |
|  | S2. Do the collected data allow to address the research questions? | Y | Y | Y | Y | Y | Y |
| **QUANTITATIVE DESCRIPTIVE STUDIES** | 4.1. Is the sampling strategy relevant to address the research question? | Y | Y | NA | Y | Y | Y |
|  | 4.2. Is the sample representative of the target population? | Y | Y | NA | Y | Y | Y |
|  | 4.3. Are the measurements appropriate? | Y | Y | NA | Y | Y | Y |
|  | 4.4. Is the risk of nonresponse bias low? | Y | Y | NA | Y | Y | Y |
|  | 4.5. Is the statistical analysis appropriate to answer the research question? | Y | Y | NA | Y | Y | Y |

**S2 Table 6. MMAT scores for studies included in sub-review question 4 - What forms of oral health care practices do adult residents of slum and other non-slum settings of LMICs engage in?**

|  | **Methodological quality** | Patel et al 2017 [20] | Osuh et al. 2022.[4] | Habib et al. 2022 [23] | Chakraborti et al 2023 [24] | Singh  et al. 2020 [40] | Gholami et al. 2012[41] | Hewlett et al. 2022 [27] | Msyamboza et al. 2016 [37] | Olusile et al. 2014  [38] | Morgan et al. 2018 [31] | Handa et al. 2016 [30] |
| --- | --- | --- | --- | --- | --- | --- | --- | --- | --- | --- | --- | --- |
| **SCREENING QUESTIONS FOR ALL TYPES OF STUDY DESIGNS** | S1. Are there clear research questions? | Y | Y | Y | Y | Y | Y | Y | Y | Y | Y | Y |
|  | S2. Do the collected data allow to address the research questions? | Y | Y | Y | Y | Y | Y | Y | Y | Y | Y | Y |
| **QUANTITATIVE DESCRIPTIVE STUDIES** | 4.1. Is the sampling strategy relevant to address the research question? | Y | Y | Can’t tell | Y | Y | NA | Y | Y | Y | Y | Y |
|  | 4.2. Is the sample representative of the target population? | N | Y | Can’t tell | Y | Y | NA | Y | Y | Y | Y | Y |
|  | 4.3. Are the measurements appropriate? | Y | Y | Y | Y | Y | NA | Y | Y | Y | Y | Y |
|  | 4.4. Is the risk of nonresponse bias low? | Can’t tell | Y | Can't tell | Y | Y | NA | Y | Y | Y | Y | Y |
|  | 4.5. Is the statistical analysis appropriate to answer the research question? | Y | Y | Y | Y | Y | NA | Y | Y | Y | Y | Y |

**S2 Table7. MMAT scores for studies included in sub-review question 5 - What is the oral health service utilization pattern of adult residents of slums and other urban residents in LMICs?**

|  | **Methodological quality** | Gholami et al. 2012 [41] | Osuh et al. 2022. [4] | Habib et al. 2022 [23] | Singh et al. 2020 [40] | Hewlett et al. 2022  [27] | Rezaei et al. 2018 [25] | Morgan et al. 2018 [31] | Olusile et al. 2014  [38] | Masalu et al.2009 [33] | Olutola & Ayo-Yusuf. 2012 [36] |
| --- | --- | --- | --- | --- | --- | --- | --- | --- | --- | --- | --- |
| **SCREENING QUESTIONS FOR ALL TYPES OF STUDY DESIGNS** | S1. Are there clear research questions? | Y | Y | Y | Y | Y | Y | Y | Y | Y | Y |
|  | S2. Do the collected data allow to address the research questions? | Y | Y | Y | Y | Y | Y | Y | Y | Y | Y |
| **QUANTI TATIVE DESCRIP TIVE STUDIES** | 4.1. Is the sampling strategy relevant to address the research question? | NA | Y | Can’t tell | Y | Y | Y | Y | Y | Y | Y |
|  | 4.2. Is the sample representative of the target population? | NA | Y | Can’t tell | Y | Y | Y | Y | Y | N | Y |
|  | 4.3. Are the measurements appropriate? | NA | Y | Y | Y | Y | Y | Y | Y | Y | Y |
|  | 4.4. Is the risk of nonresponse bias low? | NA | Y | Can't tell | Y | Y | Y | Y | Y | Can’t tell | Y |
|  | 4.5. Is the statistical analysis appropriate to answer the research question? | NA | Y | Y | Y | Y | Y | Y | Y | Y | Y |
| **QUALITATIVE STUDIES** | 1.1. Is the qualitative approach appropriate to answer the research question? | NA | NA | NA | NA | NA | NA | NA | NA | NA | NA |
|  | 1.2. Are the qualitative data collection methods adequate to address the research question? | Y | NA | NA | NA | NA | NA | NA | NA | NA | NA |
|  | 1.3. Are the findings adequately derived from the data? | Y | NA | NA | NA | NA | NA | NA | NA | NA | NA |
|  | 1.4. Is the interpretation of results sufficiently substantiated by data? | Y | NA | NA | NA | NA | NA | NA | NA | NA | NA |
|  | 1.5. Is there coherence between qualitative data sources, collection, analysis and interpretation? | Y | NA | NA | NA | NA | NA | NA | NA | NA | NA |
