# Supplementary material for: Systematic review of oral health in slums and non-slum urban settings of Low and Middle-Income Countries (LMICs): Disease prevalence, determinants, perception, and practices
Source: PLoS One. 2024 Nov 8;19(11):e0309319. doi: 10.1371/journal.pone.0309319 (PMC11548750; doi:10.1371/journal.pone.0309319)
Supplement: Attachment — Submitted filename: Response to Reviewers.docx [file pone.0309319.s005.docx]

25 March 2024

Academic Editor

PLOS ONE

**Point-by-Point Response to points raised by the Academic Editor and Reviewers**

Manuscript title: ***Systematic review of oral health in slums and non-slum urban settings of Low and Middle-Income Countries (LMICs): disease prevalence, determinants, perception, and practices.*** Manuscript submission Ref. ID [PONE-D-23-35856] - [EMID:8649ee26466d444c]

Thank you for your kind review of our manuscript, your acknowledgment of its merits, and for providing us with comments to further improve it. We have carefully considered the two reviewers’ comments and questions and have revised our manuscript accordingly. This is to submit a revised version of the manuscript that addresses the points raised in the peer review process. We provide here a point-by-point response to the reviewers’ comments and questions (italicised and in blue fonts) below and include the location of the changes from the tracked changes copy of the revised manuscript.

Thank you

Sincerely

Dr. Mary E. Osuh on behalf of all authors (Manuscript submission Ref. IID [PONE-D-23-35856] - [EMID:8649ee26466d444c]

**Reviewer 1:**

Review Comment-: “*This systematic review evaluated the disease prevalence, determinants, perception, and practices of oral health in slum and non-slums urban settings of LMICs. My comments and concerns are below:*

*• Authors should provide the specific definition for key terms used throughout the study. These include definitions for:
 o Slum and non-slum settings
 o Urban and rural settings”*

Response: The authors note this comment with appreciation.

We have now included the definition of slum and non-slum urban settings as well as Urban and rural settings in the body of the text. These can be found in the following locations:

Slum/ non slum urban settings: Page 5 lines 104 – 112 under the subtitle Introduction

Rural/ Urban settings: Page 11 lines 261 – 263 under the subtitle- Strategy for data presentation and synthesis

• *Narrative review work mentioned in the introduction should be referenced. If not published, authors should declare that the work is “unpublished.”*

Response: The authors note this comment with appreciation

The narrative review work mentioned is not published and so has been designated so. This appears on Page 5 lines 114 – 116.

The statement now reads:

*Prior to this study, we had conducted a preliminary narrative review (unpublished) and it revealed very few studies conducted on oral health in slum settings globally…*

• *What classification criteria (e.g., World Bank etc.) was used to identify the countries that formed LMICs in the study? How many countries in total? What year was the classification criteria introduced/used by the authors?*

Response: The authors note this comment with appreciation

The classification criteria used to identify countries that formed LMICs in this study and the year the classification criteria were introduced have now been included in the text. It can be found on Page 7; lines 157 – 162.

The statement reads: *For the definition for the Low and Middle Income countries (LMICS) we adopted the LMIC search filter developed by Cochrane which compiled their filter based on Word Bank Group classification system for 2021 [11]. In the classification system, countries were divided into four groups based on their gross national income (GNI) per capita: high, upper-middle, lower-middle and low. Therefore, the upper-middle, lower-middle and low-income countries are classified as LMICs [11].*

• *Abbreviations for less common electronic databases like CRD DARE and ELDIS should be accompanied with full meanings.*

Response: The authors note this comment with appreciation.

We have now accompanied the acronyms with their full names within the text and can be found in Page 7, lines 145 – 150.

It reads: …. *the following databases (MEDLINE (Ovid); Embase (Ovid); PubMed; Centre for Reviews and Dissemination (CRD) Database of Abstracts of Reviews of Effects (DARE); Electronic Development and Environment Information System (ELDIS); Essential Health Links; Health InterNetwork Access to Research Initiative (HINARI); African Index Medicus (AIM); Bioline International) in April 2020 [3] and updated in June 2023.*

• *The PECO framework referred to that guided the inclusion of the study should be clearly stated as sub-headings for clarity.*

Response: The authors note this comment with appreciation

We have now stated clearly the PECO framework that guided the inclusion and included as a subheading for better clarity. These changes can be found in Page 8 Lines 170 – 176.

• *Was a mixed-methods synthesis approach used for study synthesis in this review?* *Since it includes both quantitative and qualitative studies, how was synthesis done?*

Response: The authors note this comment with appreciation

We expected that qualitative studies would be found mainly for the review sub-question of “attitudes, perception, and belief about oral health status”, and therefore adopted a narrative synthesis approach for the systematic review for synthesising the predominantly descriptive and quantitative evidence with the flexibility to accommodate qualitative evidence. As only one qualitative study was eventually identified and included in the review, we used a convergent/concurrent design as described by Fetters et al. by collecting and analysing data from quantitative and qualitative studies separately in parallel, and for integration of the evidence to take place during the reporting and interpretation stage of the review.

We have now revised the text to reflect the method used in the synthesis and the reference is also provided.

The statement now reads:

*“A narrative synthesis approach was adopted given the predominantly descriptive and quantitative nature of the evidence while maintaining the flexibility to accommodate qualitative evidence [16]. For the review sub-question “attitudes, perception, and belief about oral health status” where a qualitative study was found, we used a convergent/ concurrent design as described by Fetters et al. [17] by collecting and analysing data from quantitative and qualitative studies separately in parallel. Integration of the evidence took place during the reporting and interpretation stages”. Available on Page 11, lines 245 - 251*

*• I see that only a study is qualitative in nature. Authors may consider excluding this study to reduce heterogeneity of study methods in the included study. Else, authors should consider the mixed methods synthesis approach which may be difficult given one qualitative study.*

Response: We thank the reviewer for the suggestions. While we agree that the inclusion of a qualitative study increases the methodological heterogeneity of evidence included in our review, qualitative study is an appropriate study design to address our review sub-question of ‘Attitudes, perception, and belief about oral health status’. As our review team included researchers with qualitative research expertise, the slight increase in methodological heterogeneity in the included studies and corresponding complexity in synthesis method did not cause any particular problems, and we believe that data from the qualitative study complemented data from quantitative studies well and have enriched the synthesis as a whole. Therefore, we do not think that it is necessary to exclude the qualitative study. We are aware of more sophisticated methods for the integration of qualitative and quantitative evidence; nevertheless, as there was only a single qualitative study, we felt that our approach to synthesising quantitative and qualitative findings through juxtaposing the data and then making comparisons and contrast at reporting and interpretation stages is sufficient for the purpose of this review while avoiding over-complicating the synthesis methods.

**Reviewer 2:**

*Review Comment-: Thanks to the authors for the effort spent researching and writing this article. However, there are many questions about the study, as follows:

- Why the systematic review was chosen as a study design? Are there other designs that are more appropriate (e.g. scoping review)? Please justify.*

Response: We have chosen the systematic review design for a couple of reasons: (1) our initial scoping of the literature indicated that the number of related studies is likely to be small; (2) we not only intended to know the volume and nature of relevant literature, but also wanted to appraise and summarise currently available evidence. A ‘standard’ systematic review is therefore more suitable than a scoping review in this context. We were aware that our research questions were relatively broad, and therefore adopted a flexible approach to assessing and synthesising evidence, e.g. by using the narrative synthesis approach and the MMAT tool which accommodates studies of different designs.

*- Please add to the introduction some definitional sentences about the slum environment in a manner consistent with the definition mentioned in the protocol published in PROSPERO.*

Response: The authors note this comment with appreciation. We have now included into the introduction, additional definitional sentences consistent with the published protocol, about the slum environment.

It reads: *The slum residents who are socially marginalised and deprived have poorer access to oral health care services, thus increasing the trend of dental diseases among them [4, 6, 7].* Page 5 Lines 112 - 114

The earlier definitional statement which had addressed the 1^st^ reviewer comment is in Page 5; lines 104 - 112

*- As for databases and literature searches, what about Scopus, Web of Science, and other databases? How is it ensured that no articles could be included in the current review in these databases?*

Response: We have conducted extensive searches using health-related databases where studies relevant to this review are most likely to be found.

In response to the peer reviewer’s comment, we have now extended our database search to include Scopus (with a limit to the subject area of dentistry) and Web of Science. Retrieved records were mostly duplicates. We found a single additional study that met our inclusion criteria and have now incorporated this study into our review. The corresponding changes have also been effected in all affected sections of our manuscript from abstract through conclusions.

*- You mentioned that you used calibration exercises to refine the screening process. Could you please explain in detail?*

Response: The authors note this comment with appreciation.

We have now expanded this statement to provide details on how the calibration exercise was done to refine the screening process. This can be found in Page 9; line 210 to 213.

The statement now reads: *Calibration exercises were conducted to ensure screening was done consistently to reduce potential errors. At the early stage of screening the reviewers compared their independent decisions and discussed and resolved inconsistencies, and refined the study selection criteria where needed.*

*- The researchers mentioned in the Materials section: "Only full-text articles written in English and publications from 2000 to June 2023 were included.". Wouldn’t it have been better to expand the scope of the search to include articles published before 2000, since the subject studied is not a recent topic, and therefore there may be articles about it within the nineties?*

Response: The authors note this comment with appreciation.

Although more studies might be located by extending the literature search before year 2000, findings from these studies would be less relevant to contemporary situation, which is our primary focus. We therefore believe that the year limitation is justifiable considering the additional time and resources that would be required. We have acknowledged this limitation.

The statement can be found in page 38; line 652-654 which reads: *This review was limited to articles written in the English language and restricted to articles published from the year 2000, hence it is possible that some literature may have been missed.*

We have also justified the reason for our time restriction in the inclusion and exclusion criteria. It reads: *Only full-text articles written in English were included. As our primary focus is on contemporary situation, we restricted the studies to only publications from 2000 …onwards*

Page 8; lines 187 - 189
