# Supplementary material for: Systematic review of oral health in slums and non-slum urban settings of Low and Middle-Income Countries (LMICs): Disease prevalence, determinants, perception, and practices
Source: PLoS One. 2024 Nov 8;19(11):e0309319. doi: 10.1371/journal.pone.0309319 (PMC11548750; doi:10.1371/journal.pone.0309319)
Supplement: Attachment — Submitted filename: Response to Reviewers.docx [file pone.0309319.s006.docx]

23 July 2024

Academic Editor

PLOS ONE

**Re: Point-by-Point Response to points raised by the Academic Editor and Reviewers**

Manuscript title: ***Systematic review of oral health in slums and non-slum urban settings of Low and Middle-Income Countries (LMICs): disease prevalence, determinants, perception, and practices.*** Manuscript submission Ref. ID [PONE-D-23-35856] - [EMID:8649ee26466d444c]

Thank you for your kind review of our manuscript, your acknowledgment of its merits, and for providing us with comments to further improve it. We have carefully considered the two reviewers’ comments and questions and have revised our manuscript accordingly. This is to submit a revised version of the manuscript that addresses the points raised in the second round of the peer review process. We provide here a point-by-point response to the reviewers’ comments and questions (italicised and in blue fonts) below and include the location of the changes from the tracked changes copy of the revised manuscript.

Thank you

Sincerely

Dr. Mary E. Osuh on behalf of all authors (Manuscript submission Ref. IID [PONE-D-23-35856] - [EMID:8649ee26466d444c]

**Reviewer #2:**

*Review Comment-: The authors have satisfactorily addressed my comments raised in the previous round of review, and I believe that this manuscript is now acceptable for publication.*

Response: The authors note this comment with appreciation.

**Reviewer #3:**

*Review comment:  The strenuous efforts of the authors to conduct this comprehensive review (Suggestion: Lines 611-612-we should avoid absolute terms like these as authors; the review looks like a bird-eye view of oral health situation in urban slums) are appreciable*.

Response: We thank the reviewer’s recognition of our efforts in the preparation of this review. The line numbers quoted in Reviewer 3’s comments above and below do not seem to correspond to the line numbers appearing in the version of the manuscript that we submitted and the PDF file created by the manuscript submission system. We have tried our best to match the reviewer’s comments with our manuscript content, but the discrepancies in line numbers made it quite challenging for us to respond to some of the comments. We would appreciate further clarifications from the reviewer or the editor in case any comments are considered to be insufficiently addressed.

*Review comment:  However, I would recommend to the authors to address three main issues: Reducing the article length as it may be a cause of boredom for the readers.*

Response: We have attempted to shorten the manuscript as the reviewer recommended. We are hesitant to remove some details as they were required to ensure completeness and transparency of reporting, including information requested by other peer reviewers. We are happy to move some content (e.g. details of quality assessment of included studies) to Supplementary Appendices if deemed necessary by the editors.

*Review comment:  In the methodology section, please bring together three screening questions (Lines 285-290), PRISMA, SWiM (for study selection Lines 213-214), MMAT (Line 199 for assessing the studies’ quality) in that order to make the things more sequential.*

Response: We are not sure what ‘three screening questions’ the reviewer was referring to. We believe that PRISMA, SWiM and MMAT are described in the most suitable places in the manuscript. These may be found under the following two sections: - Quality assessment (Page 10; Lines 226 - 239); and

- Strategy for data presentation and synthesis (Page 11; Lines 241 – 260)

*Review comment:  Discussion from Lines 512-582 takes the form of results which should be avoided.*

Response: Thank you. We have revised the text to minimise repetition of findings already presented in the Results section. We have also moved some texts to the Results section where they are more suitably located.

For example: the statement “*Various cleaning implements are deployed for mouth cleaning, notable among which are charcoal and “miswak” in Tanzania [33], cotton wool, chewing stick, salt and water only in Nigeria [38, 46], charcoal, sand, snuff powder, “neem”, twang in India and Tehran [30, 37, 41]*” was moved from the discussion section to the Results section (Page 29; Lines 485 – 488)

*Review comment:  I would suggest that it should mainly take up:*

*The salient review’s findings and their implications for the policy makers/ planners and researchers.*

Response: We believe these are covered throughout the discussion.

*The limitations.*

Response: These have been covered in the “Study limitations and strengths” section.

*The impact of the changes in the registered protocol, if any, should be mentioned.*

Response: We have added the following text: “we made some changes to our original protocol as reported in S2 Appendix. These changes allowed us to be more inclusive of potentially relevant literature given the paucity of studies conducted in slum settings, while being unlikely to introduce particular bias to the review.”

The statement may be found on Pages 39 and 40; Lines 690 – 692.

*Age group distribution; and decayed, missing and filled components of dental caries-implications???.*

Response: We have added the following text: “Dental caries affected all ages but were noted to increase with higher WHO index age categories. The values for the “Missing” component of the DMFT were usually the highest, followed by the “Decayed” component. The values for the “Filled” component were the lowest in all measures. These reflected the lack of oral health surveillance and access to dental services, leading to ongoing decay and loss of teeth before treatment could be obtained, highlighting the need for better access to dental healthcare in LMICs and particularly in slum setting In future population-based oral health surveys, a second caries-measuring tool – the Significant Caries (SiC) index – might be used together with DMFT to reflect the situation of the most caries-exposed individuals [47]. Information thus provided would enable countries to direct resources to the groups that are worst affected [47, 48].

The statement may be found on Pages 34 – 35; Lines 556 – 565.

*Gingivitis/ bleeding gums (**reversible and self-manageable conditions). The* *need for oral health education should be recommended).*

Response: We have added the following text: Among the sub-categories of periodontal disease, bleeding gum/ gingivitis formed the highest prevalence in most countries. The fact that these conditions are reversible and self-manageable has implications for targeted oral health education among affected population groups [50].

The statement may be found on Page 35; Lines 567 – 570

*The use of fluoride, explored in very few studies, should be stressed upon.*

Response: We have added the following text: Access to fluoride is crucial in preventing dental diseases [51, 52], yet there is a sparse of studies on populations' exposure to fluoride from sources like toothpaste, food, water, and applications in the LMICs. This information is necessary for understanding oral health risks and planning public health interventions effectively.

The statement may be found on Page 35; Lines 631 – 635

*Review comment:  Structural barriers to the availability, accessibility and use of dental services (publicly funded/ private, insurance etc.) should be brought to planners' attention.*

Response: Thank you. We have highlighted this in the discussion. (Page 38; Lines 652 – 655)

*Review comment:  The last three are related to lines 114-116 which need to be rephrased as well.*

Response: We are unclear about what the reviewer is referring to here due to the mismatch in quoted line numbers compared with our manuscript.

*Review comment:  In addition, in Line 192, exposure/ outcome (the terms more related to the experimental studies) are confusing???*

Response: (From Line 167, page 7) Our understanding is that PICO framework is more related to experimental studies where the ‘I’ stands for intervention. PECO framework is a well-recognized framework that is suitable for the formulation of research questions that explore the environmental impact on health and related outcomes (for example, see Morgan et al. Identifying the PECO: A framework for formulating good questions to explore the association of environmental and other exposures with health outcomes. Environ Int 2018;121(Ot 1):1027-1031, <https://www.ncbi.nlm.nih.gov/pmc/articles/PMC6908441/> ). As our review explores potential impacts of living in slums on dental disease and oral health practices, we believe it is a suitable framework to use.

*Review comment:  Lines 193-194/ 230-232: Who trained the reviewers and how???*

Response: We have now explained this, thank you. (Page 10; Line 222)

*Review comment:  Words to replace: Lines 246-accessed or assessed? Lines 402-403 regular dental visits or irregular ones???*

Response: Thank you. We have changed ‘accessed’ to ‘assessed’ (Page 12; Line 282). The statement concerning regular visits to the dentist (Page 25; Lines 437-439) was based on findings from Costa et al. (presented in Table 3), in which a greater prevalence of high caries severity was found among those who frequently visited the dentist (prevalence ratio [PR] = 1.18; 95%CI: 1.07 to 1.30) in comparison to those who did not make regular visits to the dentist. We believe this could be explained by reverse causation (higher severity of dental caries leading to more visits to the dentist) and therefore have changed our wording from ‘risk factors for dental diseases’’ to ‘factors associated with dental diseases’ (Lines 437-439) to avoid giving out the impression that the listed factors necessarily ‘cause’ dental diseases.
